# Correlates of Vocal Tract Evolution in Late Pliocene and Pleistocene Hominins

**DOI:** 10.1007/s12110-025-09487-9

**Published:** 2025-04-17

**Authors:** Axel G. Ekström, Peter Gärdenfors, William D. Snyder, Daniel Friedrichs, Robert C. McCarthy, Melina Tsapos, Claudio Tennie, David S. Strait, Jens Edlund, Steven Moran

**Affiliations:** 1 Speech, Music & Hearing, KTH Royal Institute of Technology, Stockholm, Sweden; 2 Institute of Biology, University of Neuchâtel, Neuchâtel, Switzerland; 3 Department of Philosophy, Lund University, Lund, Sweden; 4 Paleo-Research Institute, University of Johannesburg, Johannesburg, South Africa; 5 Senckenberg Centre for Human Evolution and Palaeoenvironment, University of Tübingen, Tübingen, Germany; 6 Early Prehistory and Quaternary Ecology, Department of Geosciences, University of Tübingen, Tübingen, Germany; 7 Linguistics Research Infrastructure (LiRI), University of Zurich, Zürich, Switzerland; 8 Department of Biological Sciences, Benedictine University, Lisle, IL US; 9 Department of Anthropology, Washington University in St. Louis, St. Louis, MO US; 10 DFG Center for Advanced Studies “Words, Bones, Genes, Tools”, University of Tübingen, Tübingen, Germany; 11 Department of Anthropology, University of Miami, Coral Gables, FL US

**Keywords:** Evolution of speech, Biological anthropology, Articulatory phonetics, Cognitive evolution, Paleoanthropology, Cooking hypothesis

## Abstract

Despite decades of research on the emergence of human speech capacities, an integrative account consistent with hominin evolution remains lacking. We review paleoanthropological and archaeological findings in search of a timeline for the emergence of modern human articulatory morphological features. Our synthesis shows that several behavioral innovations coincide with morphological changes to the would-be speech articulators. We find that significant reductions of the mandible and masticatory muscles and vocal tract anatomy coincide in the hominin fossil record with the incorporation of processed and (ultimately) cooked food, the appearance and development of rudimentary stone tools, increases in brain size, and likely changes to social life and organization. Many changes are likely mutually reinforcing; for example, gracilization of the hominin mandible may have been maintainable in the lineage because food processing had already been outsourced to the hands and stone tools, reducing selection pressures for robust mandibles in the process. We highlight correlates of the evolution of craniofacial and vocal tract features in the hominin lineage and outline a timeline by which our ancestors became ‘pre-adapted’ for the evolution of fully modern human speech.

## Toward an Integrative Account of Hominin Vocal Tract Evolution

Much remains unknown about the selection pressures and sequence of events that facilitated the evolution of speech in hominins. Some aspects of these events may be traced via studies of comparative facial morphology. Humans have shallow, vertical faces, mandibles and teeth reduced in size compared to apes and other hominin species (Wrangham, 2009; D. Lieberman, 2011; Puts et al., 2012; Lordkipanidze et al., 2013; Katz et al., 2017; von Cramon-Taubadel, 2017; Lacruz et al., 2019; Zollikofer et al., 2022), and a tongue and supralaryngeal vocal tract (SVT) remarkably distinct from those of extant non-human primates (Negus, 1949; P. Lieberman, 1984, 2012; Crelin, 1987; Studdert-Kennedy, 1998; Takemoto, 2008; de Boer & Fitch, 2010; D. Lieberman, 2011; Ekström & Edlund, 2023a). The result of these restructurings of *Homo sapiens*’ craniofacial anatomy represents the creation of one of the most derived and most phonetically efficient in existence (Carré et al., 2017; Lindblom, 1983; MacNeilage, 1998).

Traditionally, literature on the subject holds that non-human primate phonetic capacities allow for a rudimentary system of speech. In this view, the fact that no such system is borne out in nature possibly reflect neural (P. Lieberman et al., 1972; Lieberman et al., 2002, 2012, 2017; MacNeilage, 1998; Jürgens, 2002; Lameira, 2017; Fitch et al., 2016; Belyk and Brown, 2017; Brown et al., 2021) and/or genetic limitations (Enard et al., 2002; Trinkaus, 2007; Fisher and Scharff, 2009). The absence of rudimentary speech in non-human primates is thus taken as evidence that other pressures drove the early evolution of speech articulators, while less articulate “early speech” may have enacted an independent selection pressure on the evolution of fully articulate speech anatomy in later human evolution, counteracting the negative side effects of increased choking risk (Negus, 1949; P. Lieberman, 1984, 2012, 2017).

However, recent advances in primate vocal control and production have disputed a number of preconceived notions. Chimpanzees exhibit several prerequisites of spoken language such as lateralization of the temporal lobes (Gannon et al., 1998) and frontal lobes (Cantalupo & Hopkins, 2001; see also Amiez et al., 2023), and can in theory produce a significant range of consonant distinctions including labial consonants such as [m], [b], and [p] (P. Ekström, 2023; Ekström et al., 2024a; Lameira & Moran, 2023; Lameira et al., 2013; Lieberman et al., 1972), dental consonants (see P. Lieberman et al., 1992), and a limited range of vowel sounds (P. Fitch et al., 2016; Lieberman et al., 1969, 1972). Primate vocal behavior exhibits a number of important features consistent with speech behavior (Table 1). As such, there is a need for an integrative account of the emergence of speech capacities that is consistent with current paleoanthropological and archaeological science. Here, we highlight a variety of changes to the hominin vocal tract, and place the emergence of these features on a common timeline with other findings.

**Table 1:** Speech-like behavior observed in great apes. Studies on non-hominids are not included. Here, we are interested in whether these behaviors are possible at all; how such behaviors were learned (e.g., through enculturation) is not emphasized (cf. e.g., Lameira, 2017; Motes-Rodrigo & Tennie, 2021)

Prerequisite	Behavior	Species	Reference
*Breath and voice control*	Volitional breath control during play with wind instruments	*Gorilla gorilla*	Perlman et al. (2012)
	Voluntary control to produce labial and lingual-labial fricative sounds	*Gorilla gorilla*	Perlman and Clark (2015)
	Inhalation to retrieve food items, exhalation to elevate a ball inside clear cylinder	*Pan troglodytes* *Pan paniscus*	Schwob (2017)
	Volitional voicing and glottal fricative sounds	*Gorilla gorilla*	Perlman and Clark (2015)
	Active voicing through a membranophone	*Pongo*^*a*^ *Pongo abelii*	Lameira and Shumaker (2019)
	Volitional utterances of the phonetic form [mama] (“mama”)	*Pan troglodytes*	Ekström et al. (2024a)
*“Speech-like” rhythms*	Open-close mandibular cycles within typical frequency window of conversational speech	*Pongo pygmaeus*	Lameira et al. (2015)
	Open-close mandibular cycles within typical frequency window of conversational speech	*Pan troglodytes*	Pereira et al. (2020)
*Contrastive vowel-like calls*	A “vowel-like space” delimited by [a]-like and [u]-like extremes	*Pan troglodytes*	Grawunder et al. (2022)
	[u]-like space observed	*Pongo pygmaeus*	Ekström et al. (2023)
*Call modification*	Individual modulates vocal output based on context	*Pan paniscus*	Taglialatela et al. (2003)
	Call structural convergence between neighboring populations	*Pan troglodytes*	Crockford et al. (2004)
	Modification of duration and number of whistles	*Pongo*^*a*^	Wich et al. (2009)
	Population-specific calls independent of genetic variation among populations	*Pongo pygmaeus* *Pongo abelii*	Wich et al. (2012)
	Hand-assisted kiss-squeaks	*Pongo pygmaeus*	de Boer et al. (2015)
	Greater vocal innovation in high-density population individual (new call types typically short-lived)	*Pongo pygmaeus,* *Pongo abelii*	Lameira et al. (2022)
*Novel calls*	At least two learned utterances, “cup” and “papa”	*Pongo*^*b*^	Furness (1916)
	A juvenile learned to reproduce words	*Pan troglodytes*	Hayes and Hayes (1951)
	Species-atypical attention-getting vocalizations	*Pan troglodytes*	Hopkins et al. (2007)
	Acquired human whistle	*Pongo*^*a*^	Wich et al. (2009)
	Mothers produce novel “come hither” calls to infants, the “harmonic uuh”	*Pongo abelii*	Wich et al. (2012)
	Offspring learn attention getting calls from mothers	*Pan troglodytes*	Taglialatela et al. (2012)
	Novel attention-getting vocalization through operant conditioning	*Pan troglodytes*	Russell et al. (2013)
	Ostensibly novel vocalization, the “wookie”	*Pongo*^*a*^	Lameira et al. (2016)
	A novel attention-getting vocalization	*Gorilla gorilla*	Salmi et al. (2022a)
	Utterances of “cup”, “papa”likely correspond to pharyngeal fricative (“c”) and labial plosive (“p” consonant sounds not observed in vocal repertoires	*Pan troglodytes*	Ekström (2023)
	Affirms conclusions by Ekström (2023), retracted tongue position inferred	*Pan troglodytes*	Shofner (2024)
	Two individuals learned to reproduce the phonetic form “mama”	*Pan troglodytes*	Ekström et al. (2024a)
*Larynx-SVT coupling*	Combination of labial fricative and vowel-like utterance with rising F_2_; “Kiss-squeaks + rolling calls”	*Pongo abelii*	Lameira and Hardus (2023)
	Disyllabic utterances	*Pan troglodytes*	Ekström et al., (2024a)
*Compositionality*	Combination of pant hoots and food calls	*Pan troglodytes*	Leroux et al. (2021)
	Combination of “Alarm-huu + waa-bark”	*Pan troglodytes*	Leroux et al. (2023)
*Audition*	Vocal tract normalization (ignored speaker sex and recognized the same vowel appropriately)	*Pan troglodytes*	Kojima and Kiritani (1989)
	Discrimination of (some) consonant phonemes, below-human-level performance overall	*Pan troglodytes*	Kojima et al. (1989)
	Above-human-level sensitivity for frequencies above 8 kHz, less-than-human sensitivity for frequencies at 2-to-4 kHz	*Pan troglodytes*	Kojima (1990)
	Synthetic speech recognition at above-chance level	*Pan troglodytes*	Heimbauer et al. (2011)
	Top-down processes facilitate speech perception when signal is presented in disrupted form	*Pan troglodytes*	Heimbauer et al. (2021)
*Recognition*	Matching conspecific vocalizations to faces	*Pan troglodytes*	Izumi and Kojima (2004)
	Better recognition for natural than synthetic speech; both above chance level	*Pan paniscus*	Lahiff et al. (2022)
	Recognition of familiar human voices	*Gorilla gorilla*	Salmi et al. (2022b)
*Neural anatomy*	Left planum temporale larger in 17 of 18 brains	*Pan troglodytes*	Gannon et al. (1998)
	Frontal lobe asymmetry (of BA44) consistent with left-hemispheric dominance	*Pan troglodytes* *Pan paniscus* *Gorilla gorilla*	Cantalupo and Hopkins (2001)
	Left-hemispheric inferior frontal gyrus activity associated with vocal production	*Pan troglodytes*	Taglialatela et al. (2008)
	Selective right-lateralized activity in posterior temporal lobe in response to some (not all) calls	*Pan troglodytes*	Taglialatela et al. (2009)
	Voluntary vocalizations associated with grey matter increases in ventrolateral prefrontal and dorsal premotor cortices	*Pan troglodytes*	Bianchi et al. (2016)

^*a*^* Hybrid*

^*b*^* Species undefined; work preceded modern species division*

## Basics of Speech Production

**Fig. 1:**
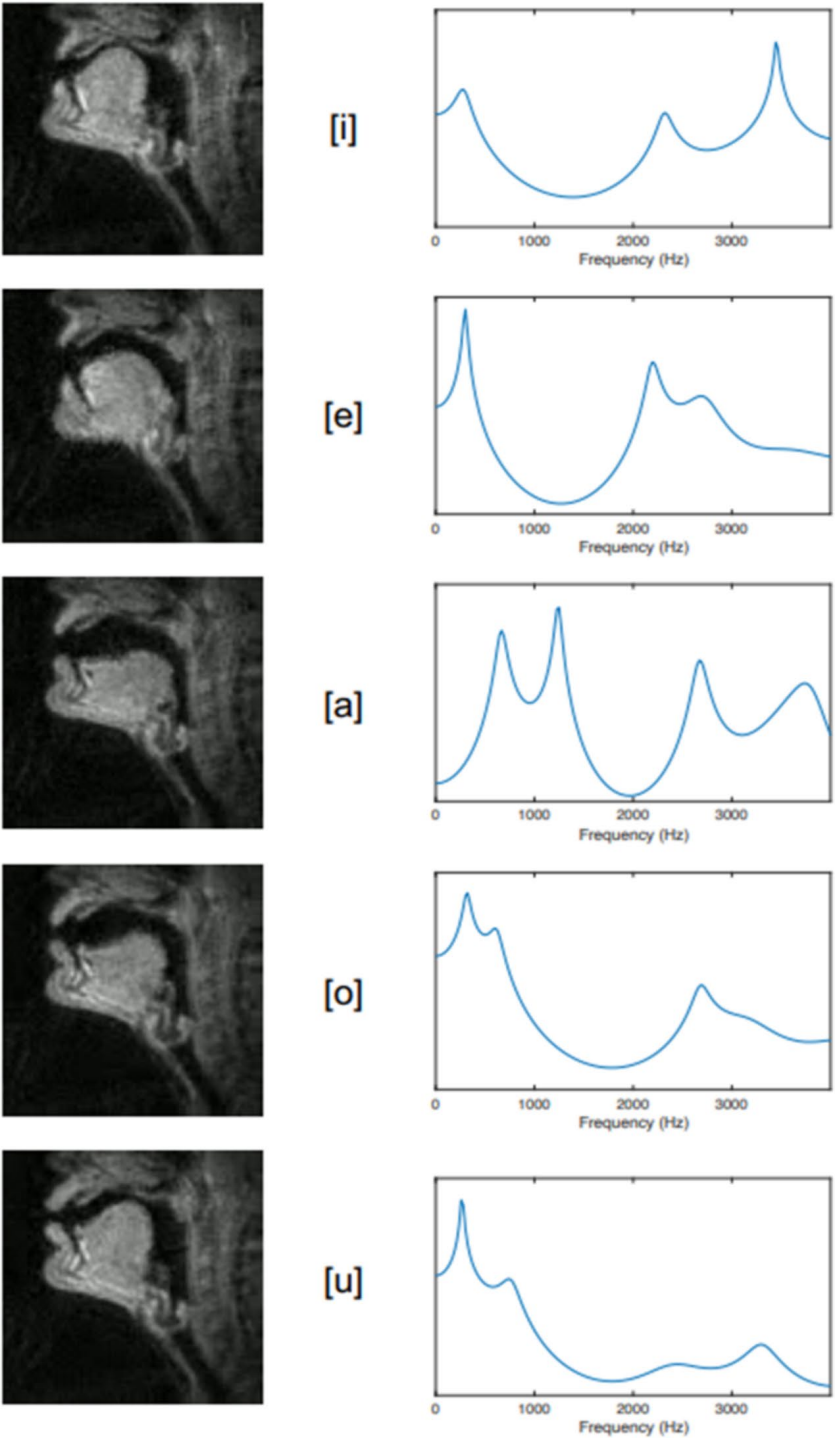
IPA Vowel chart. The front-to-back and close-to-open dimensions denote stereotyped tongue position and degree of stricture, respectively

**Fig. 2:**
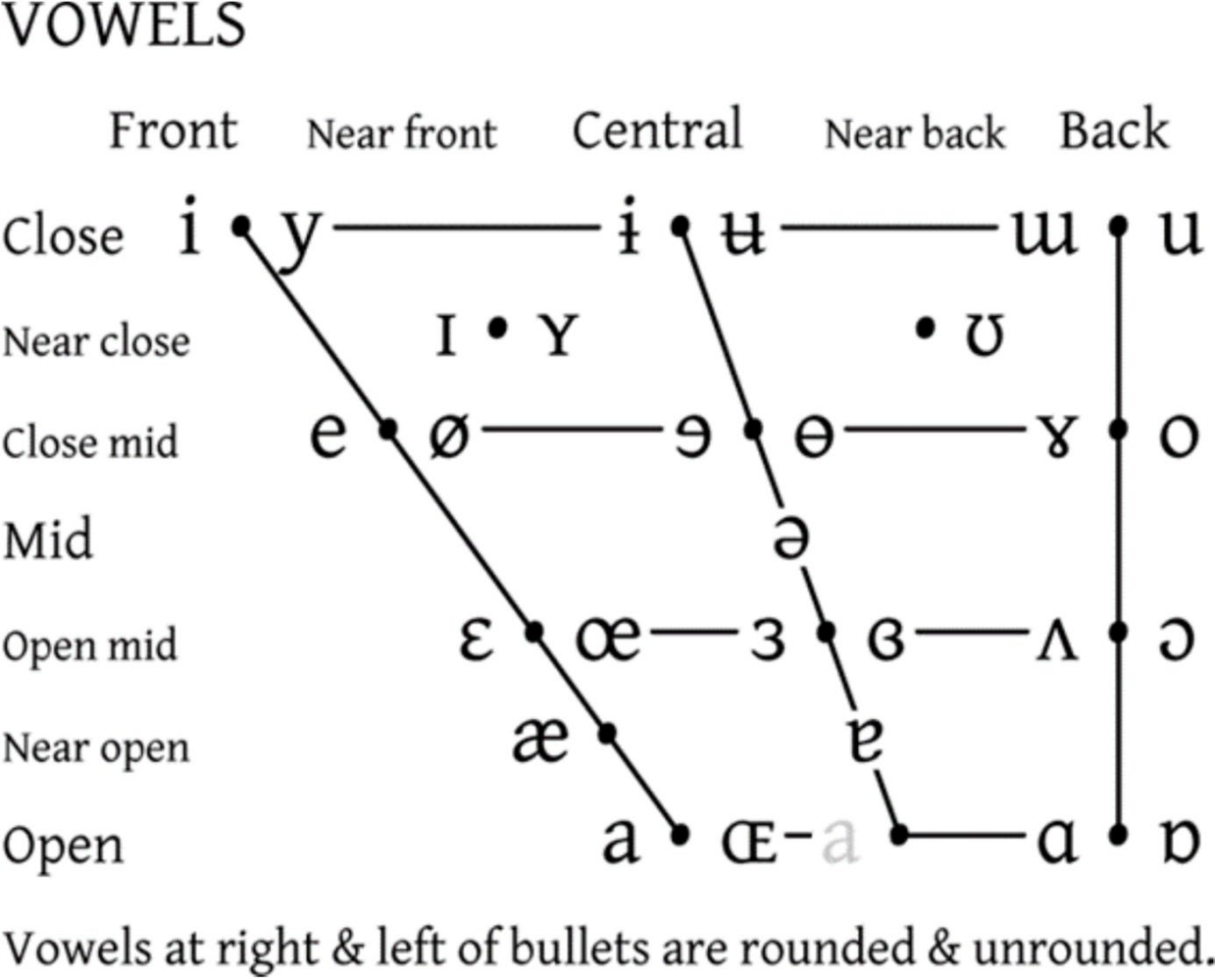
Articulatory configurations (left), and filter functions (right) for vowel tokens /i e a o u/ as produced by an adult male German speaker

Human articulate speech is a combined respiratory, phonatory, and supralaryngeal articulatory series of actions. Pulmonic airflow from the respiratory organs causes controlled vibration of the vocal folds in the larynx; the resulting voice “source” is “filtered” (Fant, 1960) by rapid and voluntary alternations between constrictions on airflow inside the SVT, resulting in controlled manipulation of resulting frequencies. For example, in producing the close front unrounded vowel [i] (the vowel in “see”), the tongue is positioned close to the hard palate in the anterior oral cavity. This creates a narrow constriction in the oral tract, shifting up the second formant frequency. Observations by Stevens (1989) showed that regions of articulatory space are stable with regard to the acoustic signal produced; for regions corresponding to “stable” vowels [a], [i], and [u], vowel quality can be achieved even when articulation is imprecise (Stevens, 1989; but see also Diehl, 1989, 2008). These vowels are also called point vowels, referring to the extremity of their articulations, as pictured in the International Phonetics Association (IPA) vowel chart (Fig. 1 and Fig. 2).

Other speech sounds are produced in different ways. For example, the voiceless velar plosive [k] (the first consonant in “cat”) involves a brief occlusion of pulmonary airflow using the tongue body followed by a rapid release burst. When coarticulated (produced as part of a sequence of speech sounds, as in everyday speaking), for example as part of a [VkV] (vowel-/k/-vowel) utterance (e.g., “iki”), the consonant imposes a brief but total suspension of voice. In humans, the ritualizing and socially deliberated and negotiated use and reuse of such intra-oral gestures form the bases for phonological systems in all the world’s spoken languages (Fant, 1960; Liljencrants & Lindblom, 1972; Moran & McCloy, 2019; Stevens, 1989). The consistent and reliable acquisition of such articulatory gestures by human infants in infancy and toddlerhood (Ekström, 2022; Kuhl & Meltzoff, 1996; Lindblom & MacNeilage, 2011; Vihman, 2014) represents a rapid transition from non-speech to speech, an expansion of combinatorial complexity far outweighing any other in nature (Corballis, 2002; Doupe & Kuhl, 1999).

The articulatory, acoustic, and perceptual structure of human speech is “chunky” by fortuitous design, largely organized into series of syllables consisting of vowel “content” couched in consonantal “frames” (MacNeilage, 1998). Articulatory gestures^1^ between two consecutive phonemes during speech production is not a one-parameter trajectory. Kinematics of a consonant–vowel syllable are more accurately described via a combinatorial score specifying a series of actions to be carried out by articulatory, phonatory, and respiratory organs. Even seemingly simple utterances are essentially multi-channel events. Take, for example, the syllable [ku] (“koo”). The velar plosive [k] is produced via the brief-but-complete occlusion (called “stop”) of airflow in the oral tract by the tongue body against the hard palate (an “occlusion”), which is then released in a “plosion” of energy. However, in [ku], lip rounding for [u] is observed progressing while closure is being executed, and the observable formant dispersion suggests that the tongue is in a back position at the moment of release. For all articulators to be in position for [u] shortly after the release of [k], rounding of the tongue and other movements must be initiated well in advance. This sequence demonstrates that movements of any two adjacent speech sounds always overlap in time, a universal principle of coarticulation.

Speech, thus, is not only a matter of control, but also the physiological elements that allows for fine-grain orientation and maneuverability inherent to continuous speech (Ekström & Edlund, 2023a; Lindblom et al., 2009; Liu et al., 2022; Öhman, 1967; Studdert-Kennedy, 1998). Research on listener perception of stop consonants (e.g., [p k t], the first consonants in “pat”, “cat”, and “tat,” respectively) illustrates that coordinated speech activity serves as identifying cues, with formant transition patterns indicating subsequent consonants (Delattre et al., 1955; Dorman et al., 1977; Kewley-Port, 1982; Liberman et al., 1967). Formant transitions in [dV] utterances illustrate this point. Like [p k t], [d] is a stop consonant. In speaking [di] (“dee”), F_2_ exhibits a telltale upward shift, while for [du] (“doo”) the transition is in the opposite direction (the transition for F_1_ is the same for both syllables). However, human vocal anatomy did not spring into existence simply “for purposes of speech” (Negus, 1949): relevant morphology evolved from pre-existing anatomical structures which themselves evolved for other functional and behavioral roles.

Evolution equipped modern humans with the capacity to produce sounds in isolation, in combination, and at variable rates (requiring anatomical evolution), the capacity for planning the production of subsequent sounds (dependent on neural evolutionary changes), and the “cultural consciousness” to enable the persistent, cumulative, and socially negotiated use of syllabic and phonemic vocal communication. To explain the richness, distinctiveness, and full extent of human speech, a combined account of anatomical, neural, and cultural evolution is not only desirable, but necessary. To this end, we trace the correlates of the evolving articulatory complex in human ancestors. Our account connects evolutionary changes in the articulatory complex and vocal tract (P. Lieberman et al., 1969, 1972, 1992; P. Lieberman, 1984, 2012; Carré et al., 1995; Nishimura, 2005; Takemoto, 2008; de Boer & Fitch, 2010; D. Lieberman, 2011; Ekström & Edlund, 2023a) to the advent of mechanical food processing (Semaw et al., 1997; Panger et al., 2002; Gott, 2002; Wrangham & Conklin-Brittain, 2003; Wrangham, 2009; Zink & D. Lieberman, 2016; Snyder et al., 2022), the cognitive faculties that made them possible (Gärdenfors & Högberg, 2017; Gärdenfors & Lombard, 2018; Lombard & Gärdenfors, 2023; Osvath & Gärdenfors, 2005; Snyder et al., 2022; Tennie et al., 2017; Vaesen, 2012; Völter & Call, 2014), and changes in brain size throughout hominin evolution (Aiello & Wheeler, 1995; Carroll, 2003; D. Lieberman, 2011; Burini & Leonard, 2018; Ponce de Leon et al., 2021; Zollikofer et al., 2022).^2^ In the following sections, we connect a variety of changes to the morphology of the articulatory complex throughout human evolution in the context of inferred changes to hominin behavior including cooking, tool use and manufacture, social behavior, and morphology. Reflecting the relative sparsity of the early Pliocene fossil record, we focus on paleoanthropological evidence from the late Pliocene and Pleistocene epoch, but where available also draw upon relevant data from extant great apes. We conclude with speculations about the origin of syllabic vocal production in the hominin lineage.

## Evolution of the Vocal Tract

The vocal tract was significantly reconfigured in hominin evolution, with non-human great apes exhibiting a different shape and position of the hyoid bone (Falk, 1975; Steele et al., 2013), and a short and narrow pharynx and expansive oral cavity (Bermejo-Fenoll et al., 2019; Negus, 1949; Sato et al., 2023), compared to those of anatomically modern *H. sapiens*, or modern humans. Anatomical reconfiguration of the vocal tract, involving expansion of the pharynx, permanent descent of the tongue root and larynx into the throat, and rounding of the tongue body has been widely regarded as an adaptation for speech (Negus, 1949; Lenneberg, 1967; P. Lieberman, 1984, 2012, 2017; Fitch, 2000; de Boer & Fitch, 2010; D. Lieberman, 2011; Ekström & Edlund, 2023a; Sato et al., 2023). Some animals like big cats also have low larynges (Weissengruber et al., 2002), but their tongues remain anchored in the oral cavity, reflecting disparate evolutionary pressures. As summarized by P. Lieberman (2006, p. 278), “A low larynx does not signify an SVT that can produce the full range of human speech”.

The derived form of the human SVT is achieved during postnatal ontogeny and human infants are born with vocal tracts resembling those of non-human primates, with a high larynx, narrow pharynx, and tongue contained in the oral cavity. Throughout early childhood, the mouth of human infants is shortened, in relative terms, through a rotation of the skeletal structure supporting the palate (D. Lieberman et al., 2000), gradual descent of the larynx and tongue root (D. Lieberman & R. McCarthy, 1999), and lengthening of the neck (Mahajan & Bharucha, 1994), ultimately achieving the roughly equally-proportioned horizontal (oral cavity and oropharynx) and vertical (pharynx) sections of the vocal tract that characterize the adult configuration (D. Lieberman & R. McCarthy, 1999; Vorperian et al., 2005, 2009; Moran et al., 2024). This configuration is dangerous in the sense that it results in an increased risk of choking during swallowing as a bolus of food passes over the laryngeal opening, which is covered by the epiglottis but not sealed off by a locked soft palate-epiglottis, the configuration in other animals. Choking on food remains a cause of death in modern human; as such, the reorganization of the hominin vocal tract would appear to reduce reproductive fitness (Palmer et al., 1992). Positive selection pressure must have outweighed negative selection for choking.

The first attempt at determining the phonetic capacities of non-human primate vocal tracts was undertaken by P. Lieberman et al., (1969, 1972), who investigated the phonetic capacities of a rhesus macaque (*Macaca mulatta*). The apparent inability of non-human SVTs to articulate “quantal” vowels (Stevens, 1969, 1972, 1989) formed a cornerstone of Lieberman’s theory of spoken language evolution (P. Ekström, 2024; Lieberman, 1984, 2012). In this view, non-human animal vocal tracts lack the capacity to produce the full extent of human speech sounds, including vowels [a] (“ma”), [i] (“see”), and [u] “true” [u] (“boot”) *in the same way as humans* – sounds found nearly universally across human spoken languages (Moran & McCloy, 2019) and held by Lieberman (1984) to be noteworthy for their articulatory and perceptual distinctiveness (Peterson & Barney, 1952; Nearey, 1978; Stevens, 1989; P. Lieberman, 1984, 2012, 2017; Friedrichs et al., 2017; Friedrichs & Dellwo, 2023; but see Diehl, 2008). Recent data has nuanced this account, though not fully refuted it. A study by Fitch et al. (2016) replicated Lieberman’s macaque study, confirming the “Lieberman account” (P. Ekström, 2024; Lieberman, 2017) and showing that macaques cannot produce the full extent of human vowel space‒even allowing for extreme contortions of the mandible and pharyngeal muscles involved in yawning (Ekström, 2024; Everett, 2017). Namely, their capacities do not include the full extent of the human vowel space.

Further, while [u]-like sounds may be approximated by other animals, including baboons (Boë et al., 2017), chimpanzees (Grawunder et al., 2022), and orangutans (Ekström et al., 2023), they cannot be reproduced identical tongue gestures as those employed by modern human speakers (P. Berthommier, 2020; Berthommier et al., 2017; de Boer & Fitch, 2010; Ekström, 2024; Lieberman et al., 1972; Nishimura, 2005; Takemoto, 2008). Boë et al. ascribed human articulation of [u] and [a] to baboons based on analogy to human speech data without reference to *in-situ* articulatory data from baboons, neglecting the possibility that such vowel-like properties likely result from species-unique constraints. Reflecting the comparative shape of human and baboon tongues and SVTs (Berthommier et al., 2017; Negus, 1949), such articulation is impossible. Rather, alternative gestures appear to explain these vowel-like properties (Berthommier, 2020).

Thus, morphology apparently prevents humanlike articulation involving deformation of the tongue body to the extent required for these vowel sounds, and recent modeling efforts suggest that the formant dispersions estimated from chimpanzee “hoo’s” may be achieved through other articulatory configurations (Ekström & Edlund, 2023b). Grawunder et al. (2022) document the occurrence of [a]-like formants in chimpanzee “barks,”, but these vocalizations are uttered with a distinctly lowered mandible beyond the range required for “true” [a], a situation unconducive to fluid coarticulated speech. In short, the occurrence of “vowel-like” formant dispersions does not necessitate (or even imply) that such utterances are produced in the same way as their seeming human language counterparts. Rather, the extreme mandible positions employed indicate that articulatory configurations are more costly in comparison.

## Late Pliocene

### Why the Long Face? The Early Hominin Vocal Tract

The nature of the hominin fossil record, in particular the lack of preservation of soft tissues including the tongue and other elements of the SVT, means that there are only hints regarding the phonetic capabilities of our fossil ancestors and close relatives (Lieberman & R. McCarthy, 2015; Clark & Henneberg, 2017). By the appearance of *Australopithecus afarensis* ~ 3.7 – 3.0 million years ago, early hominins were obligate bipeds. The transition to upright walking may also have facilitated the evolution of fine breathing control using the thoracic muscles at some unknown point after 1.6 million years ago, as inferred by differences in vertebral canal proportions between *Homo erectus* and *H. sapiens* (Hewitt et al., 2002; MacLarnon, 1987; MacLarnon & Hewitt, 2004). Once the head was no longer tethered to the thorax (Bramble & D. Lieberman, 2004) the hominin neck could more freely vary in relation to ecogeographic parameters; in the words of Bramble and Lieberman (2004, p. 350), “cranially oriented glenoid cavities (present in *Australopithecus*) … would tend to … minimize axial rotation of the head” (see also Sato et al., 2023). There is incremental evidence that australopiths possessed laryngeal air sacs (Alemseged et al., 2006), a configuration that has been argued to impede speech (de Boer, 2012). However, in the “articulator-call” acoustical scheme noted by Grawunder et al. (2022), chimpanzees are shown to produce [a]-like and [u]-like extremes delimiting their vowel-like space in continuity with human speech, suggesting that air sacs could have a limited effect on speech production.

Following works by Laitman (Laitman & Heimbuch, 1982; Laitman et al., 1979), Crelin (1987, 1989) performed a series of investigations on cranial and speech-centric morphology, arguing that the skulls of australopiths and *Homo habilis* were – with regards to speech capacities – essentially “apelike”, whereas *H. erectus* skulls were intermediate in form between the earlier australopiths and *H. sapiens*. According to this view, the basicranial prerequisites for speech arose late during hominin evolution, and only recent hominin lineages would have evolved the capacity to produce the full range of human speech sounds. Crelin’s interpretations with regards to the basicranium are challenged by more recent works (Gunz et al., 2020; Ponce de Leon et al., 2021) that are agnostic as to the relationship between basicranial anatomy and vocal tract proportions. Crelin (1987) argued that the full extent of modern human speech capacities likely had evolved recently in human evolution. Later developments revealed a number of problematic assumptions (discussed in Sect. "Neanderthal speech and the late origins of the modern human vocal tract") implemented in the Crelin reconstructions, ultimately rendering these efforts ambiguous. Nonetheless, there is ample evidence of substantial evolution of several speech articulators throughout hominin evolution, most prominently involving the jaw and other craniofacial features.

During the course of evolution, the face of *H. sapiens* has undergone a rapid reduction (D. Katz et al., 2017; Lieberman, 2011; Lordkipanidze et al., 2013; von Cramon-Taubadel, 2017; Zollikofer et al., 2022). While many non-human mammals are prognathic, with faces protruding anterior to the anterior cranial fossa and the frontal lobes of the brain, the modern human face is almost completely orthognathic (flat) and the anterior-most end of the vocal tract (i.e., the lips) protrudes only marginally anterior to the anterior cranial fossa. This is significant for two reasons. First, reduction in the size of the oral cavity and oropharynx roughly equalizes the lengths of the horizontal and vertical segments of the vocal tract. Second, prognathic animals achieve variable vowel qualities by alternately “flaring” and elongating their vocal tracts, movements accomplished by lowering and raising the mandible (Shipley et al., 1991; Schön Ybarra, 1995; P. Lieberman, 2012; Schötz, 2020; Goncharova et al., 2024; Ekström et al., 2024b). As such, the loss of prognathism meant an effective loss of such “coasting effects” of elongated horizontal vocal tracts.

Retraction of the face below the frontal lobes of the brain, flexion of the basicranium, and shortening of the face and underlying naso- and oropharynx (D. Lieberman et al., 2002; Trinkaus, 2003) create a “spatial packing problem” on the underside of the cranium, eventually necessitating laryngeal descent, untethering the hyoid from the lower border of the mandible. Importantly, the oral cavity of non-human great apes, per se, likely does not impose limits on possible speech production; it is interaction with other elements that create meaningful pressures on any speech behavior. The shortening of the oral cavity, descent of the tongue root into the throat (along with permanent descent of the larynx), and expansion of the pharyngeal cavity effectively unlocks the extremes of phonetic potential exploited today universally by human speakers (P. Lieberman et al., 1972, 1992; Laitman, 1983; Carré et al., 1995, 2017; de Boer, 2010; P. Lieberman, 2012). The concomitant rounding of the tongue also makes possible the fine distinction between various articulatory targets (Ekström & Edlund, 2023a; Gay, 1974; Lindblom, 1963; MacNeilage, 1998; Öhman, 1967; Studdert-Kennedy, 1998) unavailable to hypothetical speakers equipped with “unconfigured” vocal tracts.

Movements of the mandible, particularly the oscillatory actions of opening and closing, influence the amplitude modulation of the speech signal, which in turn shapes the syllabic organization of speech. Consequently, the mandible has often been regarded as a “serial organizer” of speech patterns (Barlow & Estep, 2006; Gracco & Abbs, 1988; Lund & Kolta, 2006; MacNeilage, 1998). In everyday speech, humans typically produce about four syllables and around 12 phonemes per second (Levelt, 1999; Poeppel & Assaneo, 2020), though even faster rates are attainable. Interesting parallels have been observed in a variety of non-human primates (Bergman, 2013; Ghazanfar and Takahashi, 2014) including great apes (Lameira et al., 2015; Pereira et al., 2020) where lip movements and smacks, resembling “speech-like rhythms” within the frequency range of 3–8 Hz, have been documented. These findings suggest that the fundamental biological basis of syllabic rhythms might share a common ancestry. A recent study by Piette et al. (2022) found that vocalization patterns across 89 masticating species predominantly manifest within this frequency range. As properties of the mandible differed vastly between species, such patterning is suggestive of broad biological mechanisms at play influencing these rhythms, beyond the specific nuances of mandible morphology. Yet, this overarching biological rhythm does not fully encapsulate its role in the temporal organization of human speech. Recent work by Friedrichs and Dellwo (2022) posits that longer mandibles in modern humans can potentially limit syllable production rates, especially under conditions necessitating rapid articulation. This might suggest that, even within the broad biological rhythm observed, the specifics of mandible morphology may impose a cap on possible syllable production rates, delineating the upper boundaries of syllable repetition. Phonetic consequences of mandibular morphology, thus, should not be overlooked.

Mandibles of extant non-human great apes have a simian shelf – a boney horizontal ridge projecting inward from the inside of the mandible, effectively thickening the bone at the lower border of the mandibular symphysis. This shelf is somewhat reduced in early hominins, but a postincisive plane is nonetheless meaningfully expressed in australopiths and early *Homo,* including *H. erectus* (e.g., Strait & Grine, 2004). P. Lieberman and colleagues (1972) argued that the simian shelf would preclude proper articulation of “true” back rounded vowel [u], which involves the creation of a narrow pocket in front of the mandibular incisor teeth. Relevant modeling suggests that apparently vowel-like vocalizations by non-human primates are produced differently than they are in modern humans (Berthommier, 2020), consistent with this idea. Australopiths possessed multiple cranial adaptations for masticating mechanically resistant foods (e.g., Jolly, 1987; Peters, 1987; Strait et al., 2009, 2013; A. L. Smith et al., 2015a, 2015b), including larger mandibles (Demes & Creel, 1988; Humphrey et al., 1999). The later reduction of this feature in *Homo* is widely considered to signify some type of shift in diet or food processing. Consistent with this idea, Stedman et al. (2004) have argued that the gene encoding the predominant myosin heavy chain expressed in chimpanzee masticatory muscles was inactivated in the lineage leading to *Homo* around ~ 2.4 mya, a change associated with reductions in size of both individual muscle fibres and the total size of masticatory muscles.

The mandibles and teeth together constitute a primary weapon for extant non-human great apes (including chimpanzees and gorillas) when engaging in confrontations and combat with conspecifics (Hill et al., 2001; Kortüm & Heinze, 2013), and in chimpanzees when hunting (Goodall, 1986; Wrangham, 1975). Reduction of the canine teeth in early hominins may denote the loss of this functional role for the mandible in australopiths and later hominins. The loss of “ape-like” mandibular robustness may have been possible in early hominins for all the reasons listed above, e.g., because much of food processing was already outsourced to the hands and tools (Schick & Toth, 1994; Toth & Schick, 2018). In a hypothetical hominin lacking behavior such as consistent use of stone tool technology, the necessity of greater masticatory forces may have prevented any loss of mandibular robustness from taking hold in earlier hominin populations. The gradual reduction of the mandible likely eased limitations on would-be syllable production rate, enforced on human ancestors and collateral relatives through the comparative robustness of their mandibles. Insofar as a gracile mandible is a pre-adaptation of modern human syllabic speech, it is reasonable to infer that changes in food processing effectively served as a pre-adaptation event (or series of events) that ultimately set the stage for its evolution.

### Early Tools of the Trade

Archaeological records suggest that while early hominins likely engaged in mechanical processing of the kind commonly found in extant non-human great apes, the invention of stone tool technology may have marked the beginning of additional cognitive evolution. Extant chimpanzees appear to plan for the future (Bräuer & Call, 2015; Mulcahy & Call, 2006; Osvath, 2009; Osvath & Osvath, 2008) and certainly make use of tools, including for food acquisition (Boesch & Boesch, 1990; Johnson-Frey, 2003), though important distinctions may yet be made with regard to such behaviors. In the words of Van Casteren et al. (2022), “for our ancestors, before the onset of cooking and sophisticated food processing methods, the costs [of chewing] must have been relatively high” (see also Schick & Toth, 1994). Because neither humans nor chimpanzees have obvious signs of dental adaptations for chewing meat specifically, Wrangham and Conklin-Brittain (2003) argue that early hominins made systematic use of meat tenderizing techniques. Even rudimentary slicing and pounding techniques would have drastic effects on chewing time, with Zink and D. Lieberman (2016) concluding that “selection for smaller masticatory features in *Homo* would have been initially made possible by the combination of using stone tools and eating meat.” This is suggestive of a meaningful relationship between the would-be speech articulators and the ecological constraints which may have limited their “pre-adaptiveness” to speech evolution. In addition, softening food via putrification and/or fermentation (Speth, 2017) could have facilitated the same changes in the chewing apparatus. These approaches are, however, significantly more difficult to track than stone tools, which is why we shall focus on such tools in this account.

Simple, putative stone tools with sharp cutting edges, as well as cut-marked bones, appear in the archaeological record approximately 3.4 – 3.3 mya in East African landscapes occupied by early hominins such as *A. afarensis* and *Kenyanthropus platyops* (Harmand et al., 2015)*.* Oldowan stone tools – consisting of simple choppers, flakes, and spheroids, the earliest stone tools to be widely accepted as such in the current literature – have been associated with various early pre-modern hominins. These tools first appear between 3.0 and 2.5 million years ago (Semaw et al., 2003; Plummer et al., 2023) and subsequently become increasingly common in the African archaeological record. It is impossible to know for certain which hominins made these tools, but representatives of *Australopithecus, Paranthropus* and *Homo* existed in Africa at the relevant times (Harmand et al., 2015; Heinzelin et al., 1999; McPherron et al., 2010).

The use of such tools for food processing may have been varied, from the cracking of nuts and hunting – as have been observed, e.g., in wild chimpanzees (Boesch & Boesch, 1983; Goodall, 1986) – to the cracking of animal bones and butchery (Boëda et al., 1999; Jacob-Friesen, 1956; Keeley, 1980). Osvath and Gärdenfors (2005) have argued that the origins of “anticipatory cognition” – the ability to mentally represent future needs – is reflected in Oldowan stone tools (see also Toth & Schick, 2018), although even earlier hominins occasionally developed similar stone technologies (Harmand et al., 2015; Lewis & Harmand, 2016; Panger et al., 2002; Semaw et al., 1997, 2003). The invention of Oldowan tools does not necessitate cultural copying of know-how (Snyder et al., 2022), suggesting that later (and perhaps much later) tool-making capacities may have been maintained and refined through cultural transmission proper.

### A Brain Made for Speaking?

Endocranial volume has increased fourfold in hominins over the past two million years (D. Lieberman, 2011; Hawks, 2011; Montgomery, 2018; DeSilva et al., 2021). Estimates for chimpanzee endocranial volume ranges from 282 cm^3^ to up to 557 cm^3^ (Herndon et al., 1999; Isler et al., 2008; Neubauer et al., 2012; Tobias, 1971; Zihlman et al., 2008). In comparison to the engineering constraints on non-human primate vocal tracts with regard to producing the full range of human speech sounds, the capacity for mapping articulatory targets may be a product of neural evolution, as evident from the modest success of non-human great apes subjected to rigorous speech (Ekström, 2023; Hayes & Hayes, 1951) and sign language exercises (Gardner et al., 1989). Apes are apparently limited with regards to learning many new vocal behaviors, even when subjected to human tutorship (Ekström, 2023; Ekström et al., 2024a; Lameira, 2017; Shofner, 2024). Australopiths may have evolved neural but not peripheral speech substrates. Compared to chimpanzees, the australopith brain was likely slightly larger, and there are signs that it is reorganized in *A. africanus* (Holloway et al., 2004; Tobias, 1968, 1971), *A. sediba* (Carlson et al., 2011) and *A. afarensis* (Gunz et al., 2020).

Unfortunately, while recent advances have allowed researchers to infer something of the presence and extent of Broca’s and Wernicke’s areas (Brodmann’s areas 44 and 45, and 22 respectively) from hominin crania (Hill & Beaudet, 2023), two main impositions pose problems for inferring their relevance to speech evolution. First, the exact contributions of increasing brain size to speech is unknown. More significantly, however, a growing body of neurolinguistics literature has deemphasized the contributions of Broca’s and Wernicke’s areas to speech, pointing instead to a crucial role for subcortical circuitry in speech production (Alexander et al., 1987; Alm, 2021; Dronkers et al., 2007; Ekström, 2022; Guenther, 2016; Hodgson & Hudson, 2018; Lashley, 1930, 1951; Lieberman, 2000; Murdoch, 2001; Pidoux et al., 2018). For example, input from the cerebellum is thought to regulate speech production, facilitating the temporal organization of speech into rhythmic utterances (Ackermann, 2008). As no trace of subcortical neurons are left in fossilized crania, such insights may be forever beyond recovery. That being said, across species, there is a strong positive correlation between sociality and (relative) brain size (Connor, 2007; Dunbar & Schultz, 2007; but see Lindenfors et al., 2021), and some have argued that primates, living in large social groups where individuals have to keep track of the identities and interactions of individuals and their kin, provided particularly fertile ground for future such social evolution (Seyfarth & Cheney, 2014; van Horik & Emery, 2011).

## The Pleistocene

### Tools and Cooking

Cooking has been argued to be a “biological trait” in modern humans (Wrangham & Conklin-Brittain, 2003). With exceptions (McCauley et al., 2020), modern hunter-gatherers are generally well-versed in creating and using fire (Gott, 2002), and all have been known to eat cooked food (Wrangham & Conklin-Brittain, 2003). Even extant non-human great apes prefer cooked food (Wobber et al., 2008), and chimpanzees have been shown to delay consumption of raw food in exchange for its cooked equivalent later in time (Warneken & Rosati, 2015). Such behavioral ubiquity across hominids suggests substantial benefits from cooked foods. Indeed, a variety of findings are suggestive of such benefits.

First, food processing by fire may serve to purify even meat scavenged from other sources; many tribes of modern hunter-gatherers obtain a substantial portion of their consumed meat from scavenging (Yellen, 1991; O’Connell et al., 1998; but see also Lupo & Schmitt, 2005). It has been argued that animal foods are a necessity of modern human diets, prior to the invention of agriculture (Larsen, 2003) some ~ 12 kya, judging by the relative caloric poverty of other available food stuff. Bone marrow, subject to less extensive bacterial growth compared with meat (A. R. Smith et al., 2015a, 2015b; see also Speth, 2017), may have provided early scavenging hominins with a source of food, even when hunting was not an option. Hominins were likely consuming meat and bone marrow as early as 2.5 mya (Blumenschine & Pobiner, 2007; Cáceres et al., 2023; Plummer et al., 2023) and more certainly by 1.9 mya (Pante, 2013; Pante et al., 2018), but occasional meat consumption likely goes back further still (McPherron et al., 2010)—not least given that apes are known to consume meat. Thus, for many scavenging species, possibly including early *Homo*, cooking may have provided a way to purify meat, which may have otherwise accumulated dangerous bacterial loads (Ragir et al., 2000; A. R. Smith et al., 2015a, 2015b; Speth, 2017). Scavenged meat may thus have provided a valid, substantial source of nutrients, even in times where active hunting was not possible. A combination of scavenging and small-game hunting may have been a likely starting point in the evolution of hunting strategies involving larger game.

Cooking may result in a substantial increase in the caloric density of foods (Wrangham, 2011; Laird et al., 2016; but see also Zink & D. Lieberman, 2016; Cornélio et al., 2016). Accordingly, living humans on raw food diets (and who thus never or rarely consume processed foods) have been reported as exhibiting lower-than-average body weight, and body mass index has been found to be inversely correlated with the proportion of raw food in the diet (Carmody & Wrangham, 2009; Koebnick et al., 1999). Lombard and van Aardt (2023) argued against this hypothesis, however. By analyzing the rich variety of plant foods available in the Klasies River area in South Africa, they found that a majority of species in the catchment area can be consumed raw. Thus, it may very well be possible to survive given such a diet (presumably including raw meat). Moreover, putrification and fermentation (neither of which requires extensive know-how) can further increase digestibility (Speth, 2017). Finally, cooking typically softens food (Wrangham et al., 2009), decreases digestion time, and increases digestibility of many different starches (Kataria & Chauhan, 1988; Sagum & Arcot, 2000; Smith et al., 2001) and plant proteins (Chitra et al., 1996).

Chimpanzees spend up to half their time awake each day masticating food stuff (Ross et al., 2009; Wrangham, 1977), while modern humans spend only around 4.7% of their daily activity doing the same (Organ et al., 2011; Zink & D. Lieberman, 2016). Based on observations of chimpanzees in the wild (Goodall, 1986; Wrangham, 1975), Wrangham and Conklin-Brittain (2003) have estimated chimpanzee caloric intake from meat at around 400 cal per hour. At the same rate, modern humans would need to spend ~ 6 h per day simply satisfying energy needs (see also Van Casteren et al., 2022). To break down hard foods, modern humans cook, ferment, putrefy, cut, slice, pound, blend, combine, chemically alter, and artificially breed various food sources. The multiple ways by which modern humans manipulate and process their food, and the timing of emergence of these behaviors, have accordingly become the focus of significant recent research.

In comparison to mechanical processing, however, the deliberate application of heat to food is found exclusively in *Homo* (Wrangham, 2009; but see Warneken & Rosati, 2015; Jacobs et al., 2021). It has been suggested that the earliest impact of cooking in the known hominin fossil record is associated with *Homo erectus* (1.9 mya), who exhibited significantly reduced teeth and mandibles compared to earlier hominins (Wrangham et al., 1999; Wrangham, 2007; D. Lieberman, 2011). This estimate is controversial, however, and, given the evidence reviewed above, unlikely. There was also a reduction of mandibular muscle myosin, oral cavity volume (Lucas et al., 2006), gut volume (particularly the cecum and colon), which resulted in faster gut passage rates (Milton, 1987; Aiello & Wheeler, 1995; Chivers & Hladik, 1984; Martin et al., 1985; Hladik et al., 1999; see also Ben-Dor et al., 2011; Zink et al., 2014; Zink & D. Lieberman, 2016; Wrangham, 2017), shorter faces relative to body size (D. Lieberman, 2011), and a substantial increase in brain size (Aiello & Wheeler, 1995; Carroll, 2003; D. Lieberman, 2011; Burini & Leonard, 2018; Clark & Henneberg, 2021, 2022). Parsimoniously, all of these changes may be related to the increasingly widespread use of techniques that eased ingestion and/or digestion. For example, the use of stone tools (for pounding, cutting, slicing, etc.), putrification, fermentation, and even retrieving naturally cooked food in the aftermath of natural fires (“fire foraging”), would to various degrees have facilitated observed anatomical changes. In a later section, we explore the impact of these developments on possible speech production capacities.

Food processing, broadly considered, includes both mechanical processing (the manipulation of would-be food stuff via cutting, slicing, etc.) and cooking – the application of heat to food (Wrangham, 2009; D. Lieberman, 2011; Zink et al., 2014; Zink & D. Lieberman, 2016; Gowlett, 2016; Speth, 2017). All such processing may be conceived as preprocessing food outside the body itself, with the benefits of increasing caloric density, decreasing time spent chewing and digesting, and consequent reduction of toxins and parasites (Stahl et al., 1984; Ragir, 2000; Wrangham & Conklin-Brittain, 2003). Mechanical processing is readily observable across extant non-human primates, and thus such manual processing also preceded cooking in evolution. While prosimians such as lemurs make extensive use of the tongue for food manipulation (Iwasaki et al., 2019), the evolution of opposable thumbs in early primates meant a functional transition toward manual dexterity and manipulation of food stuff actively using the hands and fingers (Dew, 2005; Heldstab et al., 2016, 2020; Pal et al., 2018; Tan et al., 2016). Among primates, humans have especially dexterous hands (with broad, fleshy fingertips and proportionally long thumbs whose muscles allow movement independent of the other digits) enabling an enhanced precision grip, a configuration that likely emerged early in the *Homo* lineage (Diogo et al., 2012; Karakostis et al., 2018, 2021; Leakey et al., 1964; Marzke & Shackley, 1986; Marzke, 1997, 2013; Susman, 1994, 1998). However, manual food processing is also evident in extant great apes, such as orangutans opening fruit (Stoinski & Whiten, 2003) and chimpanzee termite-fishing (Sanz et al., 2009). The complexity of such processing techniques pales in comparison with that of Pleistocene hominins, however. Specifically, at some point after 2.0 million years ago, and certainly by 1.7 million years ago, more refined Acheulian tools in the form of hand-axes and large bi-facial cleavers appear in the African archaeological record. It is not possible to ascertain what populations created these tools, but their production is generally attributed to *H. erectus* (Jurmain et al., 2005; Keeley, 1980; Rose & Marshall, 1996) before the transition into the Acheulean technological niche (overview in Toth & Schick, 2018).

While the timing of the origin of cooking is subject to great dispute, it is typically assumed to coincide with control of fire (implying fire *making*). Archaeologically-based estimates for this event based on earth-ovens found at various archeological sites across Europe and the Middle East date from ~ 250 kya (Brace, 1999; James et al., 1989), 400 kya (Barkai et al., 2017; Roebroeks & Villa, 2011; Shimelmitz et al., 2014), and 780 kya (Goldberg et al., 2023; Walker et al., 2020; Zohar et al., 2022), whereas estimates based on the appearance of *H. erectus* range as early as 1.9 mya (Wrangham, 2009; Wrangham et al., 1999). More recent dates are archaeologically well-supported, while earlier estimates are based on rare (Roebroeks & Villa, 2011) and/or localized phenomena (Shimelmitz et al., 2014; Zohar et al., 2022).

Others have suggested, partly based on widespread anatomical differences present already in early-Pleistocene *Homo*, place the control of fire around 1 mya (Berna et al., 2012; Fernández-Jalvo et al., 2018) or even 1.5 mya (Hlubik et al., 2019; see also Wrangham, 2017). However, it is difficult to exclude natural fires as potential sources (e.g., Roebroeks & Villa, 2011). The earliest use of fire may not have necessitated control of the element and may be invisible to archaeological inquiry. Early *Homo* may have made use of accidental or natural fires (Gowlett & Wrangham, 2013) and even extant chimpanzees live and forage in the presence of recurring natural fires (Pruetz & Herzog, 2017; Pruetz & LaDuke, 2010). Finally, it has been argued that Neanderthals may have lived for generations without fire in times and places absent frequent natural fire (Abdolazhzadeh et al., 2022). Overall, thus, cooking likely played a major role in human evolution, but may have done so only at a relatively late date (cf. Roebroeks & Villa, 2011; Shimelmitz et al., 2014).

### Emergent Social Consciousness and Culture

One particularly salient marker of organization of primate social systems is sexual dimorphism, the phenomenon in which males and females of the same species exhibit different sizes and/or shapes. Effectively, across non-human primates, where there is strong competition for mating access to females, male dimorphic traits are exaggerated (Plavcan, 2001). In the polygynous *Pongo* and *Gorilla* genera, males may be more than twice the size of females, while in monogamous gibbons (with reduced male-male competition), the two sexes are essentially monomorphic. Chimpanzees, living in multimale-multifemale societies, exhibit a male–female body mass ratio of ~ 1.3:1, while modern humans exhibit a ratio of ~ 1.15:1 (Dixson, 2008). The evolution of this relationship in ancestral hominins, however, is not straightforward.

While there is broad agreement that australopiths exhibited strong body size dimorphism beyond the levels seen in extant chimpanzees, the australopith condition is atypical. Normally, across primates, the relative size of canine teeth constitutes a strong marker of sexual dimorphism in a species (Plavcan and Schaik, 1992; Lee, 2005). However, australopiths likely exhibited a highly “unusual combination” (Lockwood, 1999, p. 98) of sexually dimorphic features characterized by low canine dimorphism and high body dimorphism, while both australopiths and early *Homo* exhibited significant male–female size dimorphism (Plavcan & van Schaik, 1997). Due to ecological changes, mid-Pleistocene *Homo* may have collaborated in food quests, rather than foraging individually (see Tomasello et al., 2012; Wrangham, 2019*).* In this hypothetical scenario, more-collaborative, less-selfish individuals may have been favored by natural selection. To this day, chimpanzees occasionally engage in hunting and meat consumption (Goodall, 1986; Wrangham, 1975), and while seemingly less common, similar observations have also been reported in bonobos (*P. paniscus*) (Hohmann & Fruth, 2007). Chimpanzee hunting is mostly selfish, however (Tennie et al., 2009), and the timing of the origin of habitual social hunting and meat consumption is uncertain.

Wrangham (2019) has argued that a process of self-domestication, including reduced aggression, an emergence of cooperative breeding, and increased self-control, likely placed selection pressure on anatomical features, both for signaling behavior and means of communication (see also Cieri et al., 2014; Thomas & Kirby, 2018; Benítez-Burraco & Kempe, 2018; but see Sánchez & van Schaik, 2019). Anatomical changes such as a narrowing of the male face have been taken as consistent with a selection pressure favoring reduced reactive aggression during the last 300,000 ky in *H. sapiens* (Gärdenfors et al., 2012; Leach, 2003; Wrangham, 2019). This time window overlaps with the estimate proposed by the McCarthy reconstructions indicating the emergence of modern human vocal tract dimensions in early *H. sapiens* (P. Lieberman & R. McCarthy, 2015) and follows immediately upon the proposed time of mutation of the Forkhead box protein P2 (FOXP2) gene by Krause et al. (2007), believed meaningful for linguistic evolution in early humans, and shared with Neanderthals, some 300–400 kya.^3^

This estimate overlaps with the proposed time span earmarked for the emergence of cumulative culture of know-how by Tennie (2023), sometime around ~ 500 kya. Tennie (2023) places the transition cultural know-how relatively late in hominin evolution, sometime around ~ 500 kya. For potential phonetic systems, this may be meaningful; while great apes have the capacity, however exceptionally, to learn novel vocalizations (Ekström et al., 2024a; Lameira et al., 2016; Russell et al., 2013; Salmi et al., 2022a; Wich et al., 2009), there is to date no strong evidence that such forms are preserved and transmitted across generations. Yet every new human infant acquires and effectively replicates the phonetic systems of their caretakers (Thelen, 1991; Vihman, 2014). As such, the potential relevance of cultural transmission in the development and maintenance of phonetic systems is an intriguing avenue for future work.

### Neurocognitive Adaptations

A notable shift in endocranial volume took place from between ~ 500 cc and ~ 750 cc in *H. habilis* and similar early *Homo* species to between ~ 900 cc and ~ 1150 cc in all but the earliest populations of *H. erectus* (Cornélio et al., 2016; Leakey, 1966; Leigh, 1992; McHenry, 1992; Pu et al., 1977). Estimates for specimens from Dmanisi, which many researchers consider to represent early *H. erectus*, possessed endocranial volumes between ~ 550–775 cm^3^—closer to values for *H. habilis* (Rightmire, 2013; Vekua et al., 2002). Another increase is observed between *H. erectus* and *H. sapiens*, with *H. sapiens* (Holloway, 1997; White et al., 2003) exhibiting an endocranial volume of ~ 1350 cm^3^. A modern interpretation of the paleoanthropological and archaeological records suggest that neural organization – not volume alone – need be taken into account when considering cognitive evolution (Du et al., 2018; Logan et al., 2018; Montgomery, 2013, 2018; Ponce de León et al., 2021; Shultz et al., 2012). Modeling efforts aimed at elucidating the temporal trajectory of hominin brain evolution by DeSilva et al. (2021) estimated a changepoint at 2.10 ± 0.07 mya, around the first appearance of *H. erectus* in the fossil record (Herries et al., 2020). This changepoint likely reflects the beginning of a trend that appears first in early *Homo* populations and then persists and becomes more evident in later populations of *H. erectus.*

This marked increase in brain size has alternately been ascribed to increased consumption of meat (Bunn, 2007; Leonard et al., 2007; Milton, 1999; Speth, 2017), tubers (Wrangham et al., 1999), and aquatic foods (Broadhurst et al., 2002) in early hominins. *H. habilis* and *H. erectus* may also have possessed lateralized frontal lobes (Cantalupo & Hopkins, 2001; Holloway et al., 2004) and larger brains in comparison to earlier hominins (Holloway et al., 2004). Yet other accounts emphasize sociality, arguing that increased social demands effectively put a premium on cognitive skills to navigate those demands (increasing brain size in the process) (Dunbar, 1998; Isler & van Schaik, 2012). Anthropological works show that consumption of cooked foods in modern hunter-gatherers ‒ like hunting itself ‒ is often communal (Hayden, 2014; Whiteheard, 2000; Jones, 2007; Dunbar, 2014), blurring the lines between accounts: access to beneficial foods may have required collaborative hunting. Genetic evidence also shows that the FOXP2, believed meaningful for language (Enard et al., 2002; Fisher & Scharff, 2009; Zeberg et al., 2024), was present in Neanderthals (Krause et al., 2007).

### Neanderthal Speech and the Late Origins of the Modern Human Vocal Tract

Contrary to popular sentiment, it has long been a consensus among researchers that Neanderthals likely possessed a form of language (P. Johansson, 2015; Lieberman et al., 1972). Pioneering research on the question of hominin phonetic capacities was conducted by Lieberman and colleagues (P. Lieberman & Crelin, 1971; P. Lieberman et al., 1972). Their work involved the reconstruction of the SVT of the La Chapelle-aux-Saints Neanderthal skull, whose phonetic capacities were inferred by use of a computer program to explore all possible vocal tract configurations that could be fit to the reconstructed basicranium and neck (Henke, 1966). From the resulting vowel space (the two-dimensional area denoting F_1_-F_2_ correlations of vowels, with extremities corresponding to articulatory extremes), the authors observed that ‒ like predictions for vocal tracts of non-human primates (P. Lieberman et al., 1969, 1972) ‒ the Neanderthal vowel space did not include the full extent of human vowels. The reason for this apparent limitation was Neanderthals’ relatively longer faces and shorter necks and pharynges, precluding fully modern human-like production. Specifically, the uniquely acoustically sensitive regions of the upper and lower pharynx could not be sufficiently navigated (Carré et al., 2017; Stevens, 1989; Wood, 1979, 1986). Boë et al. (1999) claimed that the conclusions of the Lieberman-Crelin efforts were that Neanderthals had been incapable of speech, but this is a misunderstanding (Ekström, 2024). In reality, Lieberman and colleagues (1972, p. 303) concluded that it was “… likely that Neanderthal man’s linguistic abilities were at best suited to communication at slow rates and at worst markedly inferior at the syntactic and semantic levels to modern man’s linguistic ability. Neanderthal man’s language is an intermediate stage in the evolution of language.” According to this view, a modern human vocal tract was necessary for the extent of fully modern human speech; Neanderthal speech, limited by a relatively short neck, would have been possible, but less efficient in comparison.

However, earlier reconstruction efforts by Lieberman, Laitman and Crelin (discussed in Sect. "Why the long face? The early hominin vocal tract") suffered from methodological issues unknown at the time. Both efforts were based on the assumption that flexion of the skull base provided a reliable basis for inferring the shape of vocal tracts. Subsequent work (D. Lieberman & R. McCarthy, 1999; Fitch & Giedd, 1999) showed that, in modern humans, the tongue, hyoid, and larynx continue to descend after the point of stabilization of basicranial flexion, placing the reliability of reconstruction efforts in jeopardy (P. Lieberman, 2007). While the methodological bases of these older reconstructions have proved unreliable, later work has supported the conclusions drawn therefrom.

More recent estimates of Neanderthal vocal tracts by McCarthy (reported in P. Lieberman & R. McCarthy, 2007, 2015; P. Lieberman, 2007, 2012) suggest that the combination of long faces and short necks could not have accommodated modern human-like vocal tract proportions necessary for the greatest range of signal variability (Carré et al., 1995, 2017). Fitting the dimensions of the human vocal tract (with a “roughly 1:1” relationship between horizontal, SVT_H_, and vertical sections, SVT_V_) to the *la chappelle*, *la ferrasie*, and *le moustier* skulls effectively places the larynx in the thorax, an anatomical configuration that does not exist in any mammal (P. Lieberman, 2012). In comparison, for all but one (*Předmostí 3*) of eight Late Pleistocene *H. sapiens* specimen examined – including San Teodoro, Cro Magnon, Dolní Věstonice III, Fish Hoek, Grotte des Enfants 6, Hotu 2, and Zhoukoudian UC 101 skulls – SVT reconstructions were found to have been confined to the neck (and not extend to the thorax). Setting aside for the moment that the exact implications of modern human-like SVT proportions may be ambiguous with regard to fluid speech, these results suggest there were likely differences in vocal tract anatomy between Neanderthals and archaeologically modern *H. sapiens*.

Further caution is warranted in interpreting this estimate. The most generous estimates from the McCarthy vocal tract reconstructions give Neanderthals a ~ 1.3:1 SVT_H_/SVT_V_ proportion. If these estimates were to fall within the extreme ranges observed in modern humans, it would be cause for disputing the relevance of SVT proportions. However, most studies investigating this relationship in modern human are small-sample works, concerned with the ontogenic growth of the vocal tract (D. Lieberman & R. McCarthy, 1999; D. Lieberman et al., 2001; Vorperian et al., 2005, 2009). Moran et al. (2024) provide measurements of SVT_H_/SVT_V_ proportions for 55 adult speakers (27 females, 28 males). Results showed proportions of 1:1 (*SD* = 0.12) for males, and 1.1:1 (*SD* = 0.12) for females (reflecting the pubertal secondary descent of the larynx in males). The most disproportionate proportions observed in the sample was an outlier female at 1.24:1. While these data fall short of the McCarthy estimates of Neanderthal SVT proportions, the relative proximity of values suggests caution is warranted. To our knowledge, there is no adult speaker data to support overlap in proportional estimates between modern humans and the Neanderthal specimen studied by R. McCarthy.

That Neanderthal phonemic space was less extensive than that of modern humans was also supported independently by Barney et al. (2012). The only work to have determined that Neanderthal phonetic space was as extensive as that of modern human was performed by Boë et al., (1999, 2002). However, this work has been refuted. The authors’ procedure involved the application of an algorithm that preserves the tongue shapes of the adult humans upon which it was based. Applying the same algorithm, any vocal tract would be shown to possess the full range of modern human speech sounds (de Boer & Fitch, 2010), and so results from these simulations are uninformative about the evolution of speech. The same authors have disputed the relevance of the “1:1 proportions” assumption (Badin et al., 2014). These data dispute the model developed by de Boer (2010) by building a model that includes the lips. These estimates remain concentrated on the possibility of production of single phonemes. As such, however, these data do not dispute other speech acoustics experiments that have reached the same conclusions independently of de Boer’s efforts.

For example, findings by Carré and colleagues (1995, 2017) were that essentially human proportions were re-invented by an algorithm set to optimize information transmission capacities of a linear 18 cm tube sequence. These experiments have been exhaustively replicated (Carré et al., 2017). Thus, even if Neanderthals were *not* precluded from the full range of speech sounds, the ability to “optimally” navigate the extent of articulatory-acoustic relationships may yet have been reduced. If so, the modern human vocal tract would indeed be uniquely well-adapted to formant-based communication. Regarding Neanderthals, the open question is whether fluid speech (not solely the species’ vowel space) is unperturbed when these dimensions are significantly disturbed from the (archaeologically modern human) norm. This more complex question has never been subjected to empirical testing. Moving forward, it will be necessary to explicitly verify any phonetic advantage to fluid coarticulated speech bestowed by a change in proportions per se.

## Putting the Pieces Together: Invention of the Syllable

There is now a range of evidence indicating extant non-human great apes vocalize voluntarily (Lameira & Shumaker, 2019; Lameira et al., 2016) and are capable of learning novel articulatory gestures (Ekström, 2023; Ekström et al., 2024a; Hopkins et al., 2007; Janik & Slater, 2000; Lameira, 2017; Salmi et al., 2022a, 2022b; Wich et al., 2009), with “syllable-like” articulatory cycles observed across primates (Bergman, 2013; Ekström et al., 2024a). While species’ anatomy apparently precludes production of the full extent of speech sounds (P. Lieberman et al., 1969, 1972; Stevens & Blumstein, 1975; Stevens, 1989; Takemoto, 2008; Fitch et al., 2016; P. Lieberman, 2017; Ekström, 2024), it evidently allows for a limited set of modern human speech sounds including rudimentary syllables (Ekström et al., 2024a). This prompts the question as to why, given articulatory possibilities seemingly sufficient for a system of syllabic “proto-speech” to evolve, no such communicative system exists in non-human great apes. To elucidate why this may be so, it is necessary to briefly consider the communicative benefits of human syllabic speech over animal calls.

The cyclical nature of syllabic speech, organized roughly as frame/content (for present purposes equivalent to consonant/vowel) units, lends itself to fortuitous “chunking” of the signal (MacNeilage, 1998), which makes it more readily appreciated and perceived by human listeners (Liberman & Mattingly, 1985, 1989; Liberman et al., 1967). This is meaningful as human systems of perception and short-term memory are limited in information storage capacity at any one time (Miller, 1956; Shiffrin & Nosofsky, 1994). The syllabic nature of natural-sounding speech counteracts and eases the perceptual and cognitive load of auditory stimulus perception, with signal predictability (Peters et al., 2016; Sussman & Gumenyuk, 2005; Wilsch & Obleser, 2016) and enculturation of speech sounds (Ekström, 2022; Liberman et al., 1967; Lindblom, 1990; McMurray, 2022; Möttönen & Watkins, 2009; Pisoni, 1971) driving reliability of perception (Table 2). The development and maintenance of systems of speech tend toward exploitation of articulatory-acoustic relationships, while subject to economic constraints of production, perception, and contrast (Liljencrants & Lindblom, 1972; Stevens, 1989). To quote Liljencrants and Lindblom (1972, p. 856), “It seems reasonable to suppose that a system [of speech sounds] which has been optimized with respect to communicative efficiency consists of [sounds] that are not only ‘easy to hear’ but also ‘easy to say’.” We might reasonably expect an early system of syllabic speech to abide by the same universal principles.

**Table 2:** Speculative relationship between evolved vocal tract morphology and their possible phonetic consequences

Epoch	Morphology	Behavior
*Extant great apes*	Robust mandible	Possibly adaptation for masticating hard foods, and used in interindividual conflicts
	Laryngeal air sacs likely a derived feature, though subject to extensive variation between species	Function likely bears relationship to social organization, as evidenced by marked sexual dimorphism of the organs in sexually competitive species, but not in multi-male-multi-female living species. Acoustic effects suggested by computational models but disputed by call acoustics data
	Fleshy lips subject to voluntary control	Lip protrusion and rounding employed analgously to that observed in speech
	Flat tongue with bunching capacities largely absent from the tongue body	Tongue retraction inferred for selections of calls, velar sounds and coarticulatory capacity likely reduced
~ *3.9‒2.9 mya*	Australopiths may have possessed laryngeal air sacs, derived hyoid morphology	Air sacs likely induce “breathiness” and possibly limit range of vowel-like qualities via the introduction of an additional low-frequency formant
	“Ape-like” basicranium	Likely bears a relationship to vocal tract anatomy
	Relatively robust masticatory apparatus	Robustness of the mandible impaired maximum syllable-per-second rate
~ *2.9‒1.7 mya*	Reduced mandible myosin-heavy fibers	Affected time spent engaging the articulatory complex through masticating
~ *1.9 mya*	Gracilization of the mandible, and retraction of the face	Retraction of the face represents a pre-adaptation for speech production anatomy. Improves maximum syllable rate
	Basicranium of *H. erectus* a midpoint between Australopith and modern human	Possible relationship to pharyngeal dimensions
	Air sacs likely lost in or prior to the common ancestor of *H. neanderthalensis* and *H. sapiens*	Loss argued to have improved speech intelligibility, though exact relevance is disputed
~ *500–300 kya*	Convincing evidence of the emergence of cumulative culture	May have been meaningful in developing and maintaining culturally pertinent aspects of phonological variation
	Mutation of *FOXP2*	Exact contribution to language uncertain but lack of gene disrupts normal language use, including oral-motor planning
~ *300‒50 kya*	Human ancestors may have achieved modern vocal tract dimensions, with uniquely orthognathic faces and expansive pharyngeal cavities	The full extent of speech capacities is achieved, possibly reflecting a novel selection pressure for improved speech communication; may also have facilitated more ready exploitation of articulatory-acoustic relationships
~ *12 kya*	Bite size configuration through the introduction of agriculture	Facilitated incorporation of labiodental phonemes into existing phonologie

The first species to have realized syllabic speech would presumably have seized on the opportunity to exploit the rapid phonetic transitions made available by labial consonant-schwa or schwa-labial consonant transitions. The argument for schwa as early syllabic “content” (MacNeilage, 1998) is two-fold. First, while a variety of primates produce vowel-like calls that overlap with distributions of modern human vowels (Boë et al., 2017; Ekström et al., 2023; Grawunder et al., 2022), these appear associated with significant articulatory effort, and as such are likely not conducive to fluid coarticulated speech (Berthommier, 2020; Ekström, 2024). Second, recent data show that formant patterns in chimpanzees producing the human word “mama” are mostly consistent with schwa (Ekström et al., 2024a). To date, this represents the only evidence of truly syllabic utterances by great apes. Unless speech was invented de novo in *H. sapiens* (a suggestion we find evolutionarily implausible), vowel sounds available to the first-ever speakers of syllabic spoken language likely did not include the articulatory degrees of freedom afforded by the modern human vocal tract, as observed across human languages today (Wood, 1979, 1986; Stevens, 1989; Lindblom et al., 1983; P. Lieberman, 1984, 2012, 2017; Carré et al., 1995, 2017; Blasi et al., 2019; Moran & McCloy, 2019; Moran et al., 2021; Sato et al., 2023; Ekström, 2024).

The ability to couple phonation to voluntary mandible movements exists in extant great apes (Ekström et al., 2024a; Lameira & Hardus, 2023), and thus likely also in early-Pliocene hominins. The syllabic utterances theoretically available to these early hominins would certainly have included labial consonant–vowel utterances such as [mama], [wawa], and [baba], and combinations such as [mawa] (Ekström et al., 2024a). However, as noted previously, australopiths may have possessed air sacs, in which case their speech would have exhibited a “breathy” quality and the resulting vowel space may have been limited (de Boer, 2012). Tentative observations by Ekström et al. (2023) suggested that orangutan phonation is characterized by seemingly incomplete glottal closure, indicating a lack of signal stability (see also Nishimura et al., 2022; Sato et al., 2023). By the emergence of genus *Homo*, air sacs were ‒ judging by the comparative shape of the hyoid ‒ completely or partially lost (P. Lieberman and R. McCarthy, 2015), freeing the signal from its possible initial breathy constraints. However, syllables such as [mə] (“muh”) offer comparatively rich linguistic possibilities, resulting from their articulatory straightforwardness (as evident from their early appearance in human phonological development), and acoustic and perceptual distinctiveness. At the moment of mouth opening, acoustic energy is redirected from the nasal cavities to the oral cavity, resulting in abrupt change in sound quality. Other labial sounds including [m], [b], and [p] are likewise acoustically distinct, and can be – and some indeed are – straightforwardly articulated with unconfigured vocal tracts such as those exhibited by non-human primates (P. Ekström, 2023; Ekström et al., 2024a; Lameira & Moran, 2023; Lieberman et al., 1992) and human infants (D. Green & Nip, 2010; McCarthy, 1946). An early system of syllabic speech may well also have exploited the apparent acoustic and articulatory distinctions between partial and complete closure of the mouth and lips (e.g., “muh”, cf. “wuh”). As to the issue of why no such system evolved in non-human great apes, we see two main possibilities.

The first possibility is that anatomical-economic costs of speaking, in combination with usefulness of then-existent repertoires of calls, may have been too great to overcome. Anatomical changes such as expansion of the epiglottic space (Sato et al., 2023) and downstream increased flexibility of the pharyngeal tract may thus have been necessary for a system of speech biomechanics to evolve (Ekström, 2024; Jürgens, 2002; Lameira, 2017; MacNeilage, 1998; Takemoto, 2008). Second, more speculatively, time spent masticating by definition precluded time spent otherwise engaged.“Freeing up” of time actively engaging the mandible and vocal tract may thus have allowed the rudimentary vocal control capabilities of non-human great apes (Ekström et al., 2024a; Lameira & Shumaker, 2019) to elaborate and experiment with articulated sounds (Dezecache et al., 2021; Ekström, 2022; Fee & Goldberg, 2011; Lameira et al., 2022; LeBlois et al., 2010; Metfessel, 1935). So established, such an ability may have allowed cultural evolution to enact novel pressures on biological and genetic evolution (Heinrich, 2016; Markov & Markov, 2020) toward clarity, consistency, and signal robustness.

We endorse a combination of models. Anatomical evolution was necessary to produce the full range of human speech sounds—possibly including its most acoustically and perceptually distinct sounds –but also to efficiently navigate the full extent of articulatory-acoustic relationships (Carré et al., 1995, 2017). We have suggested that the anatomy co-opted for speech in human evolution co-evolved with adaptations to dietary change. A dietary shift toward processed (and ultimately cooked) food coincides in the paleoanthropological record with a general reduction in craniofacial size in late australopiths and early *Homo*, illustrating that human ancestors became increasingly “pre-adapted” for speech. The “outsourcing” of food ingestion and digestion (e.g., initially to hand, tool and/or fermentation etc., and later to fire) by ancestral hominins may have facilitated the reduction and subsequent maintenance of various would-be articulatory morphological elements, including the mandible and midface, facilitating more extensive, elaborative vocal-articulatory communication. With less time spent masticating food, early speakers may have been free to experiment with basic tenets of speech (Planer and Sterelny, 2021). *H. erectus* used stone tools for mechanical processing of food. Behavioral outsourcing of ingestion and digestion may have in turn facilitated both the gracilization of the mandible and subsequent growth of the brain in the *Homo* lineage. Truly cumulative culture may have emerged by the mid-Pleistocene some 500–300 kya, in archaic *Homo*, whereas a fully modern human vocal tract may have emerged as late as 100 kya (P. Lieberman & R. McCarthy, 2007, 2015; P. Lieberman, 2012).

Our tentative view integrates existing and new perspectives on anatomical, neural, social, and cultural evolution. Once established, a rudimentary system of speech sounds may subsequently have enacted novel selection pressures, preserving a novel mutation of the FOXP2 gene variant in Neanderthals and *H. sapiens* (Krause et al., 2007; Kuhlwilm, 2018; Zeberg et al., 2024) and a unique selection pressure for articulate speech in Upper Pleistocene early *H. sapiens*. Following Negus (1949) and Lieberman and colleagues (1972), this last push toward modern articulatory morphology may thus have been driven by pressure for clearer, more robust speech. It will be necessary that future work moves away from static vowel production (P. Berthommier, 2020; Boë et al., 2017; Fitch et al., 2016; Lieberman et al., 1969, 1972) and toward a model capable of explicating the relevance of comparative articulatory morphology for the rapid and fluid production of syllabic speech (Carré et al., 2017; Lindblom, 1983; Lindblom & MacNeilage, 2011; MacNeilage, 1998; Studdert-Kennedy, 1998). Any account of the evolution of speech or phonetic capacities must ultimately reconcile with the enormous suite of changes that characterized the evolution of our unique species. A synthesis of paleoanthropological, archaeological, and phonetic evidence indicates that co-evolution of body-external food ingestion and digestion techniques (including stone toolmaking and butchery), emergence of culture, and increased social consciousness all preceded (and coincided with) the evolution of a modern human vocal tract.

## Data Availability

Data sharing is not applicable to this article as no datasets were generated or analyzed during the current study.

## Footnotes

1 Note that throughout this text, we use the terminology of phonetics, such that *gesture* refers to movements of articulators affecting the speech signal, not to manual movements or facial expressions.

2 Readers of this text may be familiar with the broader literature on the evolution of language, where a prominent debate concerns the ostensible “manual”, “vocal”, or “multimodal” origins of language. In the final section, we speculate on potential applications for this broader literature. However, we emphasize that this paper is not primarily concerned with the evolution of language or speech – but with the emergence of the human articulators themselves and their apparent correlates in hominin evolution.

3 To date, the oldest available hominin genetic data are from Middle Pleistocene hominins, approx. 400 ky old (Meyer et al., 2016). As such the mutation of the gene likely followed the evolution of most major vocal tract morphological adaptations discussed in our account. In addition, we reiterate that our work is only tangentially related to language evolution per se. Involvement of FOXP2 in speech may involve facilitating general planning of speech-related motor movements (Morgan et al., 2023). However, its expression has not generally been associated with morphological elements involved in speech production. As such, we limit our inclusion of this literature to the date of the gene’s mutation as generally argued in current literature, and as a potential adaptation (“correlate”) and factor facilitating the emergence – or modification – of speech production capacities in the Upper Pleistocene.
