# Correction: Correlates of Vocal Tract Evolution in Late Pliocene and Pleistocene Hominins

**DOI:** 10.1007/s12110-025-09501-0

**Published:** 2025-10-10

**Authors:** Axel G. Ekström, Peter Gärdenfors, William D. Snyder, Daniel Friedrichs, Robert C. McCarthy, Melina Tsapos, Claudio Tennie, David S. Strait, Jens Edlund, Steven Moran

**Affiliations:** 1 Speech, Music & Hearing, KTH Royal Institute of Technology, Stockholm, Sweden; 2 Institute of Biology, University of Neuchâtel, Neuchâtel, Switzerland; 3 Department of Philosophy, Lund University, Lund, Sweden; 4 Paleo-Research Institute, University of Johannesburg, Johannesburg, South Africa; 5 Senckenberg Centre for Human Evolution and Palaeoenvironment, University of Tübingen, Tübingen, Germany; 6 Early Prehistory and Quaternary Ecology, Department of Geosciences, University of Tübingen, Tübingen, Germany; 7 Linguistics Research Infrastructure (LiRI), University of Zurich, Zurich, Switzerland; 8 Department of Biological Sciences, Benedictine University, Lisle, IL US; 9 Department of Anthropology, Washington University in St. Louis, St. Louis, MO US; 10 DFG Center for Advanced Studies “Words, Bones, Genes, Tools, University of Tübingen, Tübingen, Germany; 11 Department of Anthropology, University of Miami, Coral Gables, FL US

**Correction to: Human Nature (2025) 36:22–69**

10.1007/s12110-025-09487-9

In this article the figure captions for Figs. 1 and 2 were inadvertently switched.

**Figure:**
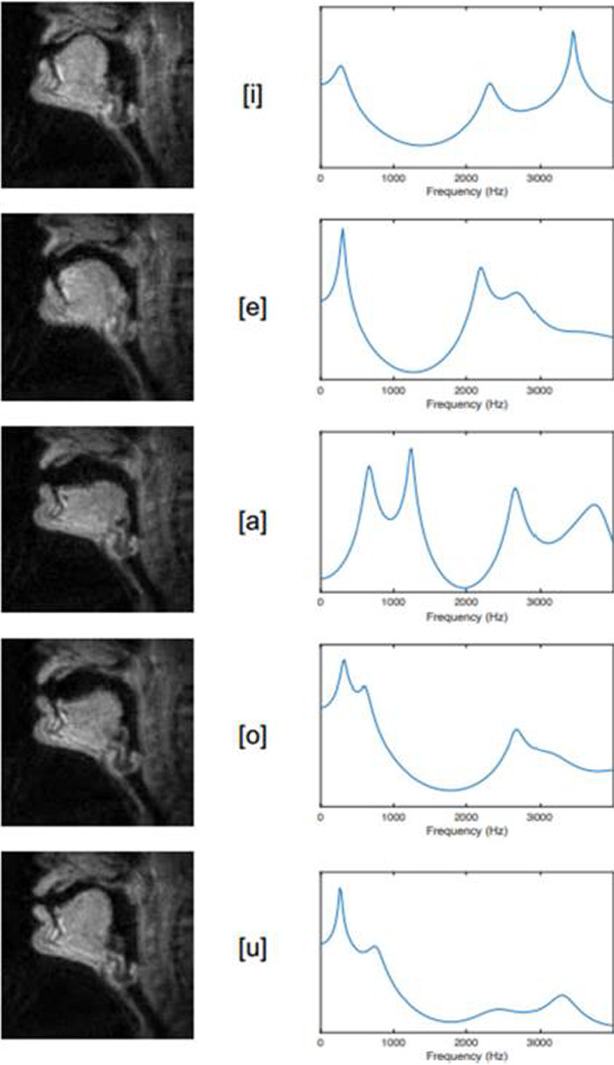

**Fig. 1** IPA Vowel chart. The front-to-back and close-to-open dimensions denote stereotyped tongue position and degree of stricture, respectively

**Figure:**
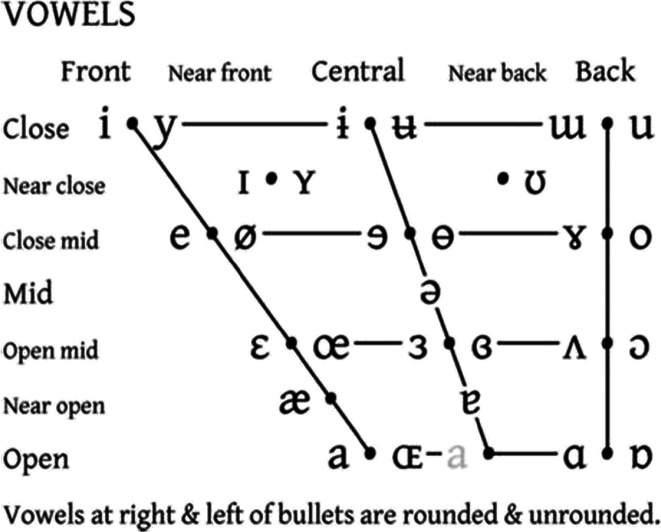

**Fig. 2** Articulatory configurations (left), and filter functions (right) for vowel tokens /i e a o u/ as produced by an adult male German speaker

The figures should have appeared as shown below.

**Figure:**
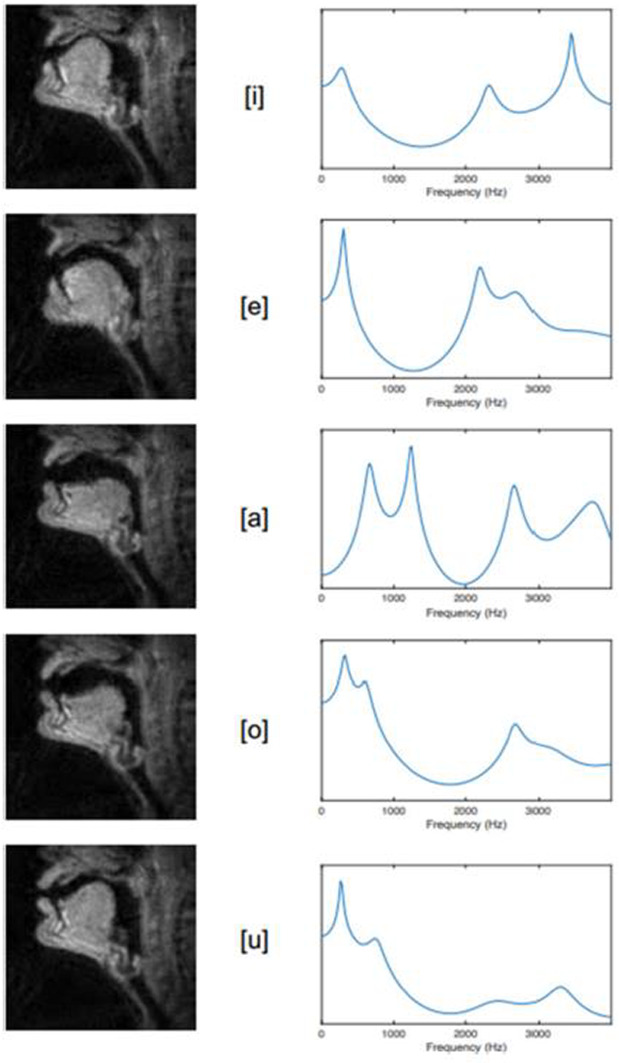

**Fig. 1** Articulatory configurations (left), and filter functions (right) for vowel tokens /i e a o u/ as produced by an adult male German speaker

**Figure:**
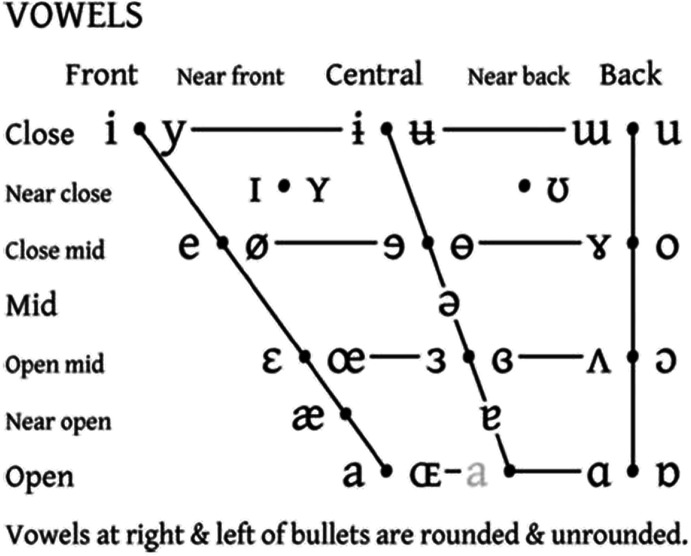

**Fig. 2** IPA Vowel chart. The front-to-back and close-to-open dimensions denote stereotyped tongue position and degree of stricture, respectively

